# A novel methodology for localizing pallidal deep brain stimulation leads

**DOI:** 10.3389/fnana.2026.1768558

**Published:** 2026-02-23

**Authors:** Benjamin Pobiel, Kevin J. O’Neill, Remi Patriat, Tara Palnitkar, Meghan E. Hill, Rachel C. Cole, Henry Braun, Stephanie L. Alberico, Biswaranjan Mohanty, Devyn Bauer, Michael C. Park, Scott E. Cooper, Jerrold L. Vitek, Noam Harel, Joshua E. Aman

**Affiliations:** 1 Department of Neurology, University of Minnesota, Minneapolis, MN, United States; 2 Center for Magnetic Resonance Research, Department of Radiology, University of Minnesota, Minneapolis, MN, United States; 3 Department of Neurosurgery, University of Minnesota, Minneapolis, MN, United States

**Keywords:** brain mapping, DBS, functional neurosurgery, movement disorders, neuromodulation, Parkinson’s disease, stereotaxy, targeting

## Abstract

**Introduction:**

Positioning of deep brain stimulation (DBS) leads is paramount for optimizing therapeutic efficacy in Parkinson’s disease (PD) and dystonia. Quantitative determination of lead position remains essential; however, current atlas-based targeting and stereotactic coordinate methods, while informative, limit patient specificity afforded by high-resolution reconstruction and introduce subjective variability.

**Methods:**

We developed a novel pipeline in Unity™ to ingest 7T MRI-based reconstructions of DBS leads within the globus pallidus internus (GPi). Using anatomical landmarks and structure-specific algorithms, the GPi was parcellated into 12 anatomically-based subregions in a semi-automated, reproducible manner. Active contact positions were localized relative to a novel coordinate system developed from a GPi-based bounding box. A novel distance-to-border metric remapped contacts onto a common atlas (PD25) for population comparison against the same contacts mapped onto a common left GPi space using mid-commissural point (MCP) coordinates (Schaltenbrand-Wahren atlas).

**Results:**

Fifteen leads from 10 PD subjects were used for ellipsoid fitting of active contact locations, resulting in an elliptical volume of 38.94 mm^3^ when using MCP coordinates, compared to a volume of 5.08 mm^3^ with our GPi-specific coordinates. The mean distance-to-ellipse centroid was 3.45 ± 1.57 mm for MCP coordinates and 2.03 ± 0.82 for our GPi-specific coordinates. Our distance-to-border remapping metric yielded mean adjustments of 0.81 mm (*y*-axis) and 1.61 mm (*z*-axis). A subset of six GPi active contacts were plotted with post-DBS motor improvement scores, demonstrating the ability to link lead location with clinical outcomes.

**Conclusion:**

Our novel software provides a quantifiable lead location with respect to the anatomical target, enhancing patient-specific lead localization by avoiding some of the pitfalls of either structure-to-atlas normalization or traditional stereotactic coordinates.

## 1 Introduction

Deep brain stimulation (DBS) of the globus pallidus pars internus (GPi) is an effective treatment for the motor signs of Parkinson’s disease (PD) and dystonia (Au et al., 2021; Groiss et al., 2009). Multiple reports have shown that the position of the DBS lead within the targeted brain region, specifically concerning the motor subregion, correlates with therapeutic efficacy in reducing motor signs (Ellis et al., 2008; Marks et al., 2009; Paek et al., 2013, Patel et al., 2015; Richardson et al., 2009; Rolston et al., 2016; Vitek et al., 2022; Welter et al., 2014). Additionally, proximity to surrounding structures (e.g., posterior limb of the internal capsule) can lead to therapeutic-limiting side effects. With continuously improved imaging resolution and understanding of surrounding neural pathways, precise localization of the DBS lead position within the targeted structure becomes vital for understanding the relationship between lead/stimulation location and clinical outcomes. By reliably normalizing lead location comparisons between subjects and across populations, additional insights can be gained into optimal surgical placement, as well as aiding clinical decision-making during lead programming.

Lead visualization and reconstruction techniques utilized in DBS procedures for PD have primarily been accomplished in single-plane slices, with nascent tools providing three-dimensional (3D) visualization (Gross et al., 2006). Software packages, such as Cicerone, Stealth, Guide XT 3.0, BrainLab (Brainlab Inc.), and Boston Scientific Programmer (Boston Scientific Inc.), allow integrating patient-specific data imaging with trajectory planning and lead position verification (Bucholz and McDurmont, 2009; Merola et al., 2021; Miocinovic et al., 2007). In all of these current techniques, localizations of planned trajectories and implanted DBS leads are frequently quantified by a mid-commissural point (MCP)-based coordinate system using the anterior and posterior commissure (AC/PC). Yet studies have demonstrated that MCP-based coordinates do not significantly correlate with motor outcome scores (Daniluk et al., 2010; Nestor et al., 2014). Additionally, MCP-based coordinates of target structures, such as the subthalamic nucleus (STN), can vary dramatically across patients. One study reports deviations of approximately 6 mm laterally, 4 mm in the A-P direction, and nearly 5 mm in the vertical direction (Daniluk et al., 2010; Nestor et al., 2014). Contributing further to variability, variations in the manual selection of AC and PC points can lead to variations in target coordinates over 1–2 mm within the STN, ventralis intermedius nucleus (VIM), or GPi (Pallavaram et al., 2008). The magnitude of this variability is certainly noteworthy, given that a two-millimeter difference in location can dramatically affect the therapeutic benefit of a DBS lead (Schrock et al., 2021). Alternatives to MCP coordinates include direct-targeting techniques involving descriptive language (e.g., “postero-dorsolateral region”), but these techniques lack a universal methodology for quantifying exactly where these subregions exist within the GPi (Au et al., 2021; Johnson et al., 2023; Vitek et al., 2022).

Mounting evidence demonstrates the need and opportunity for improved precision in our understanding of lead locations and the implications of these locations on clinical outcomes (Abosch et al., 2010; Duchin et al., 2018; Lenglet et al., 2012; Patriat et al., 2018). Recent 7 Tesla (7T) structure segmentation and functional parcellation have enabled patient-specific lead position reconstruction and surgical planning (Patriat et al., 2018). A precise, intra-structure position for optimal placement would be an additional step toward improving patient care through increased specificity during lead reconstruction.

We present a semi-automated method created in the Unity development environment using a combination of boundary detection and anatomical inputs to construct anatomical parcellations, with an intra-GPi coordinate system for precisely localizing and describing the structure-specific and structure-based position of DBS lead contacts. Our methodology describes the position of DBS lead contacts with respect to a specific targeted structure of interest rather than external anatomical landmarks or common atlases, while providing a translation from our coordinate system back to MCP coordinates for stereotactic targeting. We have also developed a border-relative adjustment function for mapping active contact locations on a population level that preserves the location of the DBS lead relative to the target structure’s borders. Given the common use of anatomical terminology for describing optimal locations for DBS lead implant and stimulation, our intent is to reproducibly identify these regions and to objectively quantify the location of leads or contacts, with the goal of clarifying current subjectivity surrounding ideal implantation sites in the GPi.

## 2 Materials and equipment

Workstations, including laptop and desktop computers running a 64-bit Windows 11 operating system, were used to both develop and run this software.

## 3 Methods

### 3.1 Statement of ethics

All patient procedures were approved by the University of Minnesota Institutional Review Board (IRB#1611M00822 and 1210M22183), with written informed consent obtained from each participant according to the Declaration of Helsinki.

### 3.2 Segmenting anatomical subregions of the GPi

**FIGURE 1:**
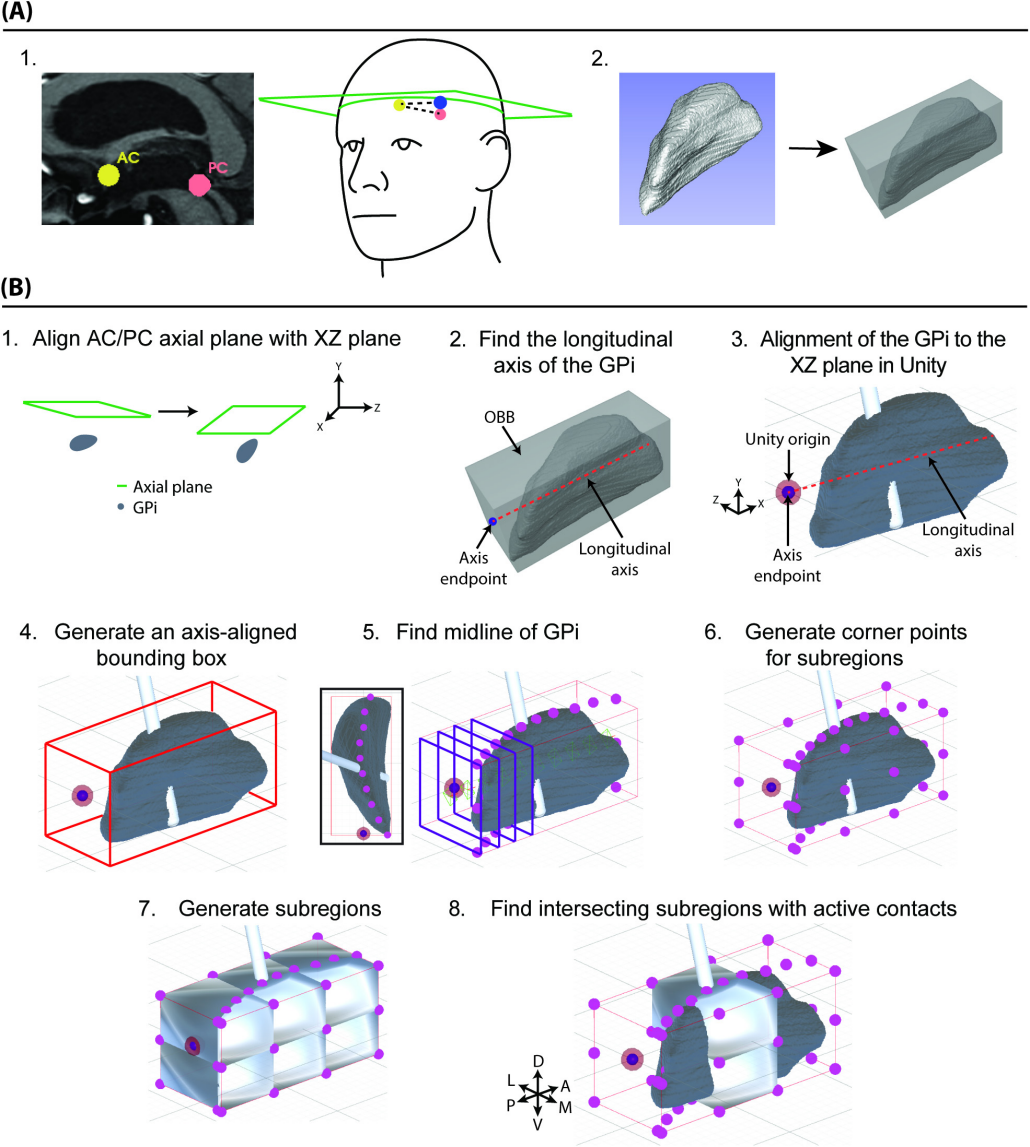
Methods for creating target-specific anatomical subregions and a target-specific coordinate system. **(A**.1**)**. The patient’s MRI was used to identify the AC and PC points (left panel, yellow and salmon) and a third point (right panel, blue) to form an axial plane (right panel). **(A**.2**)** The 3D model representing the segmented GPi (left panel) is exported as a 3D mesh and then analyzed to find the optimal bounding box (OBB, right panel). **(B**.1**)** The AC-PC axial plane (green outline) is aligned with the XY coordinate plane within the Unity development environment. **(B**.2**)** The OBB is used to generate a longitudinal axis for the GPi. **(B**.3**)** The longitudinal axis is then used to align the GPi to the XZ plane within Unity. **(B**.4**)** An axis-aligned bounding box (AABB, red outline), aligned to the coordinate axes in Unity, is generated around the GPi model. **(B**.5**)** YZ planes are drawn along the long axis of the GPi, and the centroid is found for each of the intersections to generate a curving midline through the GPi. **(B**.6**)** Points around the AABB are generated, dividing the GPi into three sections along its long axis (posterior, central, anterior), and two sections along its dorsal-ventral axis. **(B**.7**)** These points are used along with the midline points to generate 12 parcellated subregions of the GPi. **(B**.8**)** By determining which lead components (contacts) collide with both the GPi model, and anatomical subregions, an anatomically-descriptive location of the stimulating contact within the GPi can be determined.

All patients underwent preoperative 7T MRI and 30-days postoperative CT scans, both with stereotactic protocol. Patient-specific models of the GPi and DBS lead were created and then loaded into 3D Slicer^1^ (Abosch et al., 2010; Duchin et al., 2018; Patriat et al., 2018; Solomon et al., 2021). Within 3D Slicer, an axial plane encompassing the AC and PC points was generated (Figure 1A.1; Fedorov et al., 2012). The 3D model representing the segmented GPi was exported as a mesh and analyzed to find the least-volume optimal bounding box (OBB), which is the minimum-volume box containing the mesh (Figure 1A.2; The CGAL Project, 2024). Points representing the AC/PC axial plane were exported from 3D Slicer and imported along with the GPi mesh and OBB corner points into the Unity development engine, which is a cross-platform graphics engine supporting 3D interactive programs, augmented reality, and virtual reality (Unity Technologies, San Francisco, CA).

The AC-PC axial plane was aligned to the XZ (“axial”) plane in Unity space (Figure 1B.1), which rigidly transformed the position and orientation of all the scene contents. A longitudinal axis of the GPi was determined using the OBB (Figure 1B.2) and then an axis-aligned bounding box (AABB) was generated around the GPi mesh, aligned to the XZ plane in Unity space (Figures 1B.3, 4). To avoid confusion from differing naming conventions in Unity versus conventional anatomical descriptions, references in this paper will refer to the X-dimension as anterior-posterior (longitudinal axis), the Y-dimension as medial-lateral, and the Z-dimension as dorsal-ventral (Figure 1B.8).

Using the AABB, the centroids of a 2D shape formed from the intersection of 10 YZ (“frontal”) planes along the long axis of the GPi mesh were found and used to create a curving axis dividing the GPi into medial and lateral components (Figures 1B.5, 6). The AABB was then split equally into dorsal-ventral halves and into thirds along the A-P axis, resulting in the GPi being parcellated into 12 subregions: two medial-lateral halves, two dorsal-ventral halves, and three sections (anterior, central, and posterior) along the long axis (Figures 1B.6, 7). Dividing into 12 subregions was subjectively determined by domain experts at our institution to give sufficient resolution when applying our methodology to current language and guidance around implantation within the GPi (Au et al., 2021; Gross et al., 2006). Meshes were then generated to enclose each of the parcellated subregions. Lastly, a collision detection algorithm was used to determine overlap between the parcellated subregions and the DBS lead components, allowing detection of which subregions contained the active DBS contacts (Figure 1B.8). Active contacts were visually inspected to confirm their intersection with both the GPi mesh and the parcellated subregions.

### 3.3 Establishing an intra-GPi Cartesian coordinate system for localizing active DBS lead contact(s)

**FIGURE 2:**
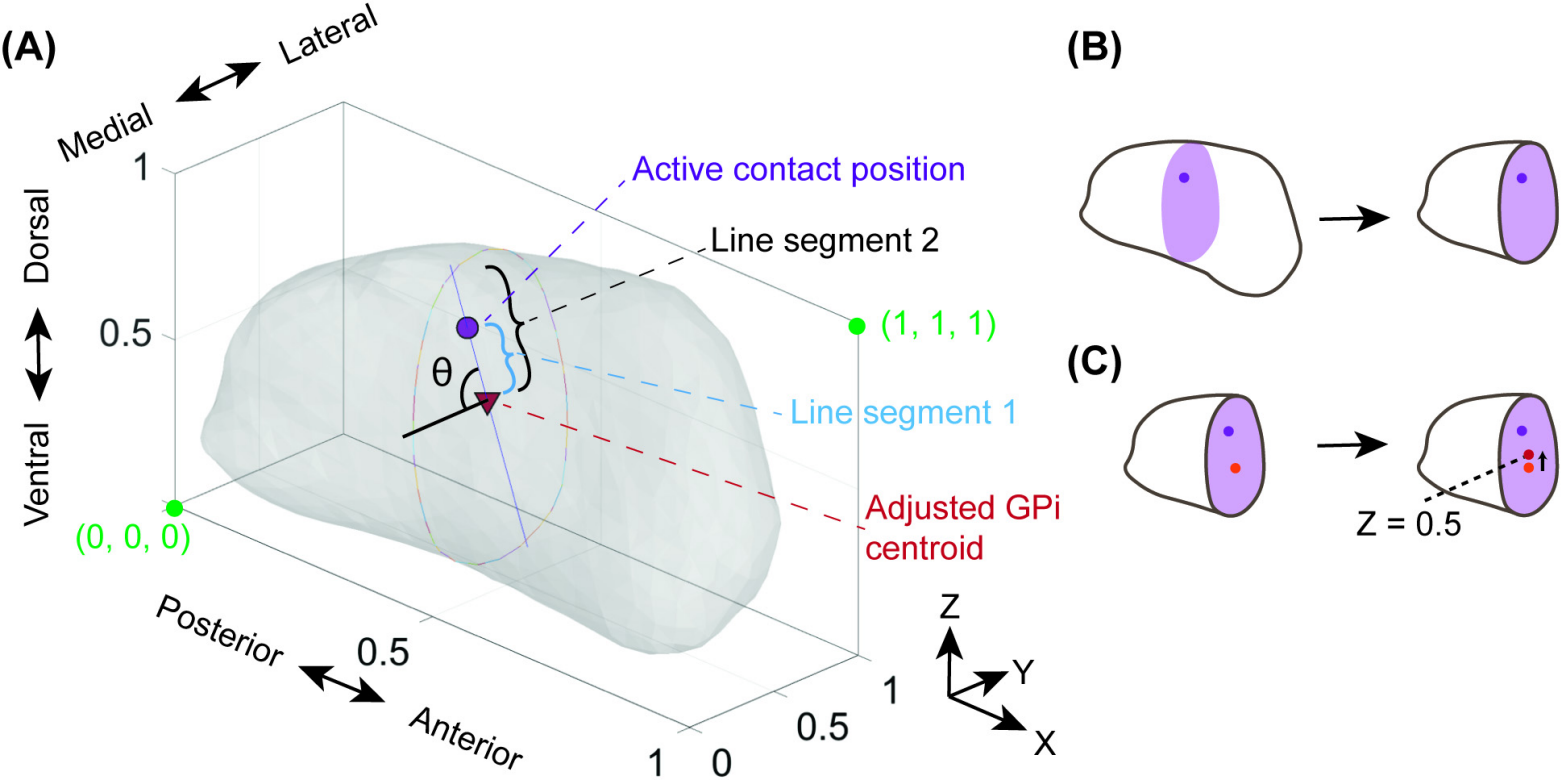
Adjusting the active contact position for population plotting. To facilitate comparisons of GPi’s with different surface topology, the relative proximity of the active contact to the surface of the GPi is determined in a systematic manner. **(A)** The relative active contact position is determined via the angle θ and the ratio between line segment 1 (blue) and line segment 2 (black). The angle θ is determined between a horizontal line extending from the adjusted GPi centroid along the *Y*-axis and line segment 1/2. **(B)** The adjusted GPi centroid is determined using a cross-sectional YZ-oriented plane (purple) at the X-position of the active contact position (dark purple point). **(C)** The centroid of this plane within the GPi is determined (orange point) and transformed up to a Z position of 0.5 (red point) in order to maintain the halfway division between the dorsal and ventral halves of the GPi previously established (Figure 1). This point is labeled as the adjusted GPi centroid.

The borders of the axis-aligned bounding box (AABB, Figure 2A) were used to generate an intra-GPi Cartesian coordinate system, with an origin point (0, 0, 0) chosen at the posterior-ventromedial corner of the bounding box, placing the point (1, 1, 1) at the most antero-dorsolateral position (Figure 2A). The selection of the origin point at the posterior-ventromedial corner enabled easy translation between the numeric coordinate to the parcellated subregions and was deemed the most transferable methodology for current 3D coordinate use (i.e., increase in coordinate value when moving medial to lateral, inferior to superior and posterior to anterior). The centroid of the active contact mesh was used to determine the relative position of the contact within the intra-GPi coordinate system. For demonstration purposes, for leads with multiple active contacts (i.e., multiple cathodes), the relative position was determined as the centroid of all the combined active contact meshes.

### 3.4 Adjusting the relative intra-GPi coordinates for population plotting and inter-patient comparison

To facilitate comparisons of patient-specific GPi’s with different surface topology, the position and proximity of the active contact to the surface of the GPi was determined in a systematic manner. We calculated a “distance-to-border” factor to facilitate this comparison. We avoided remapping the GPi mesh to prevent relative movement of the active contact position with respect to the 12 parcellated regions (Figures 2A, B). To determine the distance-to-border factor, a point representing the previously determined centroid of the active contact was plotted in the Unity scene (Figure 2B, purple dot). The GPi was then sliced in the YZ (coronal) plane along the long-axis (*X*-axis), at the X position of the active contact (Figure 2B, purple plane). The GPi centroid of this slice was then determined (Figure 2C, orange dot). An “adjusted GPi centroid” (Figure 2A) was then determined by translating the original GPi centroid in the vertical (*Z*-axis) direction until it was located at the dorsal-ventral midpoint of the GPi within that same YZ plane (Figure 2C, red dot).

Next, a line was drawn from the adjusted GPi centroid to the point representing the centroid of the active contact. The distance of this line was represented as line segment 1 (Figure 2A). A second line, colinear with line segment 1, was then drawn from the adjusted GPi centroid to the border of GPi. The distance of this second line was defined as line segment 2. A horizontal line was extended medially (-y direction) from the adjusted GPi centroid. The angle created between line segments 1/2 and the horizontal line was defined as theta (θ, Figure 2A). The ratio of the distance magnitudes for line segment 1 and 2 (line segment 1: line segment 2) along with the angle θ were then used to translate patient-specific locations into a generic GPi for population comparison, in this case, using the PD25 atlas (University of Minnesota, 2024; Xiao et al., 2015, 2017).

### 3.5 Comparing mid-commissural-based coordinates of the active contact to novel intra-structure coordinates

**FIGURE 3:**
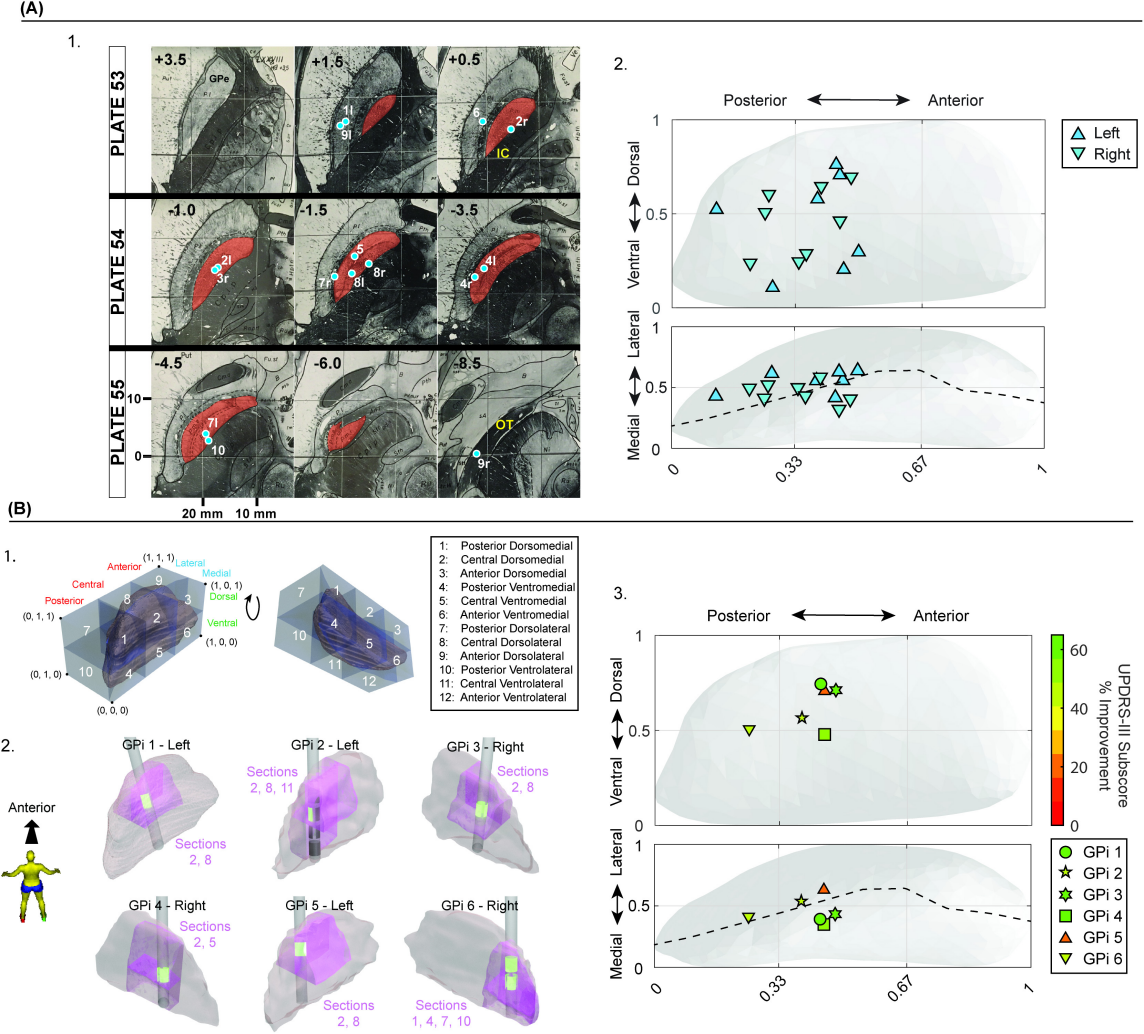
Capabilities of novel localization methodology with comparison to MCP coordinates. **(A**.1**)** Active contact positions were determined in MCP coordinates and mapped onto the Schaltenbrand and Wahren atlas. **(A**.2**)** The same active contact positions were determined using our intra-structure coordinates system and mapped onto the PD25 atlas. **(B**.1**)** Legend showing each parcellated subregion and its anatomical position. The legend has been rotated around a horizontal axis in the 2nd image on the right, to show the positioning of anatomical parcellations 11 and 12. **(B**.2**)** Patient-specific lead positions as determined algorithmically by the software created in this study. Pink regions represent the intersection between the parcellated anatomical subregion(s) and the GPi mesh in which the lead active contacts (bright green) lie, illustrating the location of the active contact within specific 3D subregions of the GPi. **(B**.3**)** The relative position of active contacts within the PD25 GPi atlas and their corresponding UPDRS improvement scores. Improvement scores were taken from baseline at the nearest available follow-up appointment, and represent scoring taken after bilateral lead implantation, except in the case of subject 3 where the scoring was taken after unilateral implantation. Active contact positions were averaged in the case of multiple utilized active contacts, such as in GPis 3, 4, and 6.

Preoperative T1 and T2 MR were registered to postoperative CT scans on the StealthStation™ S8 Surgical Navigation Systems for determining the position of active contacts (Medtronic plc, Dublin, Ireland) (Supplementary Material Section 1.1–2) These MCP coordinates were then mapped onto the Schaltenbrand-Wahren atlas (Figure 3; Schaltenbrand, 1977). To compare variability of contact mapping between the two methods (MCP versus intra-GPi coordinates), an ellipsoid volume and distance-to-centroid measures were calculated. Using a mean volume and estimated dimensions of GPi (Patriat et al., 2018), intra-GPi coordinates were denormalized and converted into absolute measures. Mean (±SD) coordinates across all patients were then calculated for MCP and intra-GPi coordinates. Standard deviations of mean coordinates were used to calculate three-dimensional ellipsoid volumes, a simplistic version of least squares ellipse fitting (Fitzgibbon et al., 1999). Additionally, the mean distance from each subject’s active contact to the group centroid was measured and averaged across all subjects using MCP and GPi-specific coordinates, separately. Ellipsoid volumes and mean distance-to-centroid were then compared between the two coordinate mapping systems.

To demonstrate the utility of our new methodology for examining the relationship between contact location and clinical outcome measures, the Unified Parkinson’s Disease Rating Scale (UPDRS) Part III motor subscores were totaled for the side of the body contralateral to the DBS lead implantation. Percent change in motor severity was calculated using post-surgery follow-up subscores (OFF-medication/ON-DBS) with respect to pre-implantation subscores (OFF-medication/OFF-DBS). Percent changes in UPDRS-III subscores were then plotted with respect to active contact location.

## 4 Results

Fifteen GPis from 10 subjects were transposed onto a left GPi and active contacts were mapped onto the Schaltenbrand-Wahren atlas (1977) using MCP coordinates (Figure 3A.1). These were compared against active contact locations adjusted via our “distance-to-border” adjusted intra-GPI coordinate system and remapped onto the PD25 atlas (Figure 3A.2; University of Minnesota, 2024; Xiao et al., 2015, 2017). The average MCP coordinates of active contacts across all subjects were *x* = 19.54 ± 2.24 mm (lateral), *y* = 4.17 ± 1.59 mm (anterior), *z* = −1.95 ± 2.61 mm (superior) (Supplementary Table 1, columns “MCP Coordinates”). The average intra-GPi-relative position coordinates using our new methodology were M-L (y) = 0.46 ± 0.18, long-axis (x) = 0.36 ± 0.11, Dorsal-Ventral (z) = 0.51 ± 0.10. Note: For intra-GPi coordinates within Unity, the medial-lateral axis is the *y*-axis, and the long axis of GPi coincides with the *x*-axis (see Section “3 Methods”).

Mean, denormalized, GPi-specific coordinates were 2.08 ± 0.81 mm (toward lateral orthogonal to the long axis), 6.1 ± 1.86 mm (toward anterior along the long axis), and 4.09 ± 0.81 mm (toward dorsal), with the origin being the most posterior ventromedial aspect of the bounding box (Figure 3B.1). MCP coordinates resulted in an elliptical volume of 38.94 mm^3^, compared to a volume of 5.08 mm^3^ when using GPi-specific coordinates. The mean distance-to-centroid for MCP coordinates was 3.45 ± 1.57 mm (range: 1.25–8.13 mm), and for GPi-specific coordinates, the mean was 2.03 ± 0.82 (range: 0.75–4.07 mm).

We then applied our “distance to border” methodology to each subject’s active contact, generating an adjusted active contact position. By definition, the methodology does not shift the position of the contact along the *x*-axis, as this is performed by scaling the bounding box along the *x*-axis (see Section “3 Methods”). Our methodology resulted in a mean adjustment of active contacts by 0.18 ± 0.13 units along the *y*-axis (M-L), and 0.20 ± 0.16 units along the *z*-axis (dorsal-ventral). A conversion to absolute measures shows adjustments of 0.81 ± 0.56 mm (range: 0.14–2.21 mm) and 1.61 ± 1.28 mm (range: 0.09–4.30 mm) in the M-L and dorsal-ventral axes, respectively. An elliptical volume calculated using the adjusted y- and z-coordinates resulted in a volume of 5.33 mm^3^, similar to the elliptical volume for unadjusted coordinates (5.08 mm^3^).

Finally, to demonstrate the utility of our new methodology for examining the relationship between contact location and clinical outcome measures, Figure 3B.2 shows the positions of 6 reconstructed DBS leads and active contacts within the patient-specific anatomical parcellations generated by our methodology. Figure 3B.3 shows the same active contacts plotted onto the PD25 atlas (Figure 3B.3) using our distance-to-border factor adjustment, along with using a color scale to represent their individual UPDRS-III subscore percentage improvements. These example data show post-DBS implantation UPDRS-III subscore improvements between 23.5% and 85.0%, with the average percent improvement in subscores being 52.7% ± 20.0% (see Supplementary Table 1 for individual percentage improvement). The mean duration between follow-up and baseline scores was 22.5 months (Stdev ± 10.3 months; range: 15.08–37.3 months). Inclusion of these data is intended to be illustrative of our methodology, rather than inferential with respect to the ideal location of targeting within the GPi.

## 5 Discussion

We have presented a novel patient-specific methodology with multiple capabilities: (1) systematically generating individualized anatomical subregions of GPi consistent with prior nomenclature; (2) three-dimensional localization of DBS lead contacts within the GPi; and (3) creating border-adjusted coordinates for group analyses of DBS lead locations. Additionally, we use a sample of PD patients with DBS lead implants to demonstrate the use of our methodology along with additional outcome variables such as post-implant changes in motor severity scores (i.e., UPDRS-III). Lead location and orientation within a target structure (e.g., GPi) may have major implications for the effects of DBS therapy due to underlying functional subregions of that structure and their respective neural pathways (van Wouwe et al., 2020). Thus, precisely quantifying lead locations is essential for interpreting activation patterns, refining surgical targeting and stimulation parameters, and ultimately elucidating the mechanisms of DBS. The methodological advancements described here provide an infrastructure for precise DBS localizations, improving atlas or direct-targeting techniques employed during surgery, and leading toward further individualized treatments both intra- and post-operatively.

Our first goal was to systematically parcellate the GPi into anatomically-based subregions using nomenclature that is consistent with previously published literature and common clinical language used when targeting the GPi (e.g., “posterior-lateral ventral” or “dorsal region”). We also considered a means for drawing some comparison to anatomical terminology used for the subthalamic nucleus (STN) given both are common targets for DBS leads in PD patients and often compared with respect to clinical outcomes. Thus, we determined subjectively that the 12 subregions presented in this methodology would have relevance to previous literature and clinical practice. Though a more precise method of localizing DBS leads was our ultimate goal, the anatomic subregions provide a systematic bridge between commonly known location terminology and novel target-relative 3D coordinates that users will not be familiar with upon initial use.

There is wide agreement that the location of the DBS lead is important for optimizing therapeutic benefit and minimizing therapy-limiting side effects. The fact that MCP-based coordinates (gold standard for target planning and reporting lead locations) often fail to predict clinical severity score improvements in the STN or GPi may indicate that a more accurate representation of the lead location is needed (Nestor et al., 2014). The lack of predictability is likely due to standardized coordinate systems not fully accounting for neuroanatomical variation between patients. We highlight this in Figure 3A.1, mapping active contacts onto the Schaltenbrand and Wahren atlas (1977), which shows three contacts in GPe, one in the optic tract, and a few contacts on or right next to the borders of GPi. We compare this to a population mapping of the same active contacts using methods presented here (Figure 3A.2) which shows all contacts fall within the borders of GPi. Differences in the reconstruction of an anatomical location of a lead can have dramatic effects on the ability to properly and efficiently program a DBS lead in the clinic or result in misinterpretation of the relationship between stimulation location and clinical outcomes. A misinterpretation of this relationship can lead to suboptimal lead placement in subsequent patients.

Coordinates from our methodology show marked reductions in the variability of reconstructed active contact locations, which is represented by a simple calculation of a standard deviation ellipsoid volume and distance to centroid measurement. While there are limitations to this analysis, this clearly highlights a reduction in location variability across a population compared to using an MCP-based atlas. Though mapping MCP coordinates directly onto an atlas likely represents the extreme difference, it does demonstrate the potential variability induced when using MCP coordinates on a standardized coordinate system compared to our patient and structure-specific approach. We interpret our methodology as a more accurate and precise representation of lead or contact locations in both individual reconstructions and border-adjusted population plots. Given the variability in the size of GPi across patients, the border-adjusted localization maintains the integrity of the location of the lead relative to the anatomical structures within and around GPi, which is vital for interpreting the relationship between the location of the lead contact(s) and stimulation-related outcomes.

Though clinical outcomes are not the focus of this paper, we show post-surgical UPDRS-III subscores with respect to stimulation location for two purposes: the first is to demonstrate our method’s capabilities, and the second is to show how stimulation in relative proximity can accompany large differences in therapeutic benefit, which could highlight the importance of precise localization. Combining our methodology with parcellations of targeted structures in functional subregions could lead to patient-specific, intra-structure (e.g., STN, GPi) targeting that considers anatomical and functional subregions for disease or sign/symptom-specific stimulation, yielding improved clinical outcomes (Patriat et al., 2018; Plantinga et al., 2018). While including functional subregions will be another important step forward, continuing to provide a classification using anatomical subregions, as shown here, may provide a reproducible bridge between classical terminology for describing lead locations and an understanding of intra-structure coordinates and subregions that enhance the precision of lead localization.

Current alternatives to atlas-based targeting include direct targeting methods, with either whole-GPi visualization, or parcellations of functional subregions or connectivity within the structure (Horn et al., 2017; Lenglet et al., 2012; Plantinga et al., 2018; Vayssiere et al., 2002). Some of these methodologies are not currently a part of the standard of care, but are likely to grow in prominence as alternatives to traditional targeting methods. We view our methodology as potentially enhancing these approaches, by providing a standardized and quantitative framework against which these models could be validated. Additionally, our software is region-agnostic. If a subregion of the GPi is deemed to be the most promising, the same principles applied here to the whole GPi could be applied to the subregion in question, albeit limited by imaging resolution or confidence in probabilistic determination of subregion boundaries.

There are multiple potential error points associated with imaging-based data. Error in our methods will be most associated with the voxel size of the image being segmented (in this case, 0.4 mm × 0.4 mm × 1 mm T2 scans) (Duchin et al., 2018). Additional error can exist from the manual segmentation process and coregistration during electrode position reconstruction. A prior study, however, has shown that intra- and inter-rater reliability is relatively high for our parcellation methodology (Duchin et al., 2018). Although data presented here were collected using a 7 Tesla scanner, our methodology could be applied to 3T reconstructions, but with likely increases in error in visualizing structure borders due to lower resolution. Still, in our experience, obtaining a low-quality image (e.g., motion artifact) contributes more to a low-confidence reconstruction than a lower-resolution image.

Some limitations of our methodology include our approach to multiple stimulation contacts, where a simple centroid was used, weighing all vertices of the different contacts equally. This may provide an incomplete picture of current delivery and the electrical stimulation field. Further iterations will likely incorporate weighting for multi-contact, complex stimulation parameters while maintaining the utility of identifying a single 3D localizing coordinate. This methodology also makes simplifying assumptions around the best way to section the GPi (such as using an AC/PC-aligned plane to determine dorsal and ventral portions of the GPi), which does provide a precise anatomical delineation but may be better represented by functional subregions within the structure, a feature likely to be incorporated.

Further improvements are planned to streamline the user experience and develop a cohesive graphical user interface (GUI). Distances to additional anatomical features can be added, such as to the accessory medullary or internal medullary lamina, relevant neuronal pathways, or functional subregions. We plan to develop the platform further to assist in quantifying intra-operative targeting decisions as well as the post-operative capabilities shown here. These additional features are, however, typically dependent on the initial imaging resolution and available sequences.

Here we have established a methodology that precisely quantifies the location of a DBS lead with respect to the patient-specific structure targeted for implant and categorically defines the location using established nomenclature through repeatable, systematic routines. We then present a methodology for translating patient-specific locations onto a standardized atlas for the purpose of population analyses, which maintains the anatomical integrity of the lead location. Furthermore, we demonstrate the important utility of associating precisely defined lead locations with clinical outcomes. Improving the predictability of this relationship is vital for preoperative target planning and for efficient postoperative programming. By building this tool in Unity, we utilize a 3D application development platform extensible to other brain regions and updatable as knowledge and guidelines about localization change. We envision a tool capable of assisting with pre-operative targeting, real-time intraoperative adjustments, and postoperative image-based programming to provide a more precise, interactive, patient-specific tool for DBS surgeries.

## Data Availability

The raw data supporting the conclusions of this article will be made available by the authors, without undue reservation.

## Footnotes

1
http://www.slicer.org
