# Supplementary material for: A novel methodology for localizing pallidal deep brain stimulation leads
Source: Front Neuroanat. 2026 Feb 23;20:1768558. doi: 10.3389/fnana.2026.1768558 (PMC12968257; doi:10.3389/fnana.2026.1768558)
Supplement: Supplementary file 1 [file Supplementary_file_1.docx]

***Supplementary Material***

# Supplementary Methods

## Structure segmentation and parcellation from 7T MRI

Patient scans were taken on a 7T MRI scanner (Magnetom 7 T Siemens, Erlangen, Germany). The scanner has a 200 T/m/s slew rate via a 32-element head array coil (Nova Medical, Inc., Burlington, MA) and is outfitted with SC72 gradients capable of 70 mT/m. The T2-weighted images were used along with a neural network, GP-net, to reconstruct GPi and GPe segmentations.(Solomon et al., 2021) These segmentations were manually corrected and reviewed by multiple domain experts. Anterior and posterior commissure (AC/PC) points were determined manually in 3D slicer, and the scene was aligned to the AC/PC axis.(Fedorov et al., 2012)

Postoperative CTs were taken at 30 days, and 3D models of the lead components (tip, shaft, and electrodes) were matched to the reconstructed electrodes and leads generated using both linear and non-linear registrations in 3D Slicer (<http://www.slicer.org>).

## Determining mid-commissural-based coordinates of the active contact

Post-operatively, a 30-day postoperative CT (CT30) was registered to the same preoperative imaging sequences used for surgical planning within the StealthStation™ for the first 6 GPis and Brainlabs for the remaining 10 (supplemental table 1). Contact locations were estimated by visualization of the artifact within the CT30. The active contact centroid was estimated considering the known dimensions of the DBS lead (i.e., contact heights, width, and distance apart). The estimated positions of the lead and contacts were then cross-checked with the original surgical trajectory/target plan and notes from the microelectrode mapping procedure for the finalized lead implant location.

## Supplemental table legends

**Supplemental Table 1.** Medtronic Stealth and Brainlabs system coordinates for active contacts relative coordinates generated by a bounding box around the GPi, along with the percent improvement in UPDRS-III. The point of origin in the Stealth system is the mid-commissural point (MCP). A positive percent change indicates a decrease in UPDRS-III scores from preoperative to postoperative scores. All UPDRS-III scores were taken after bilateral implantation, except for subject 3, where the patient had only a unilateral implant at the time of scoring.

# Supplementary Figures and Tables

**Supplemental Table 1.** UPDRS-III motor sub-score improvement and positional data from anatomical sectioning, MCP coordinates, and intra-GPi relative coordinate determination.

| GPi/Side | Subject | Intersecting sections | MCP coordinates (from Stealth* and Brainlab) | | | Intra-GPi relative coordinates | | | Percent UPDRS-III sub-score improvement |
| --- | --- | --- | --- | --- | --- | --- | --- | --- | --- |
|  |  |  | X (ML, mm) | Y (AP, mm) | Z (DV, mm) | X (+X is ant.) | Y (+Y is lat.) | Z (+Z is dors.) |  |
| 1/Left | 1 | 2, 8 | -20.58* | 5.78* | 1.17* | 0.44 | 0.70 | 0.37 | 85.0% |
| 2/Left | 2 | 2, 8, 11 | -17.16* | 4.89* | -1.03* | 0.39 | 0.57 | 0.55 | 42.1% |
| 3/Right | 2 | 2, 8 | 15.49* | 5.24* | 0.78* | 0.48 | 0.65 | 0.39 | 66.7% |
| 4/Right | 3 | 2, 5 | 16.87* | 4.06* | 0.72* | 0.45 | 0.49 | 0.31 | 57.1% |
| 5/Left | 4 | 2, 8 | -20.81* | 4.54* | -3.28* | 0.45 | 0.67 | 0.61 | 23.5% |
| 6/Right | 4 | 1, 4, 7, 10 | 22.51* | 2.93* | -3.95* | 0.25 | 0.52 | 0.48 | 41.7% |
| 7/Right | 5 | 11 | 18.84 | 5.93 | -1.38 | 0.50 | 0.31 | 0.67 | N/A |
| 8/Right | 6 | 2, 8 | 20.90 | 6.71 | 0.06 | 0.40 | 0.66 | 0.60 | N/A |
| 9/Left | 7 | 5, 8, 11 | -19.57 | 4.03 | -4.34 | 0.46 | 0.23 | 0.51 | N/A |
| 10/Right | 7 | 4, 10 | 22.48 | 2.59 | -2.28 | 0.21 | 0.32 | 0.53 | N/A |
| 11/Left | 8 | 10 | -19.19 | 3.03 | -2.32 | 0.27 | 0.10 | 0.63 | N/A |
| 12/Right | 8 | 4, 5, 10, 11 | 15.99 | 4.73 | -2.06 | 0.36 | 0.30 | 0.41 | N/A |
| 13/Left | 9 | 7, 10 | -22.04 | 0.27 | -8.63 | 0.12 | 0.50 | 0.47 | N/A |
| 14/Right | 9 | 7 | 21.61 | 5.1 | 1.41 | 0.26 | 0.58 | 0.60 | N/A |
| 15/Right | 10 | 4, 5, 10, 11 | 19.07 | 2.70 | -4.13 | 0.34 | 0.32 | 0.54 | N/A |

Abbreviations: ML, medial-lateral; AP, anterior-posterior; DV, dorsal-ventral; MCP, mid-commissural point; GPi, globus pallidus internus; UPDRS, Unified Parkinson’s Disease Rating Scale

# Supplementary References

Fedorov, A., Beichel, R., Kalpathy-Cramer, J., Finet, J., Fillion-Robin, J.-C., Pujol, S., Bauer, C., Jennings, D., Fennessy, F., Sonka, M., Buatti, J., Aylward, S., Miller, J.V., Pieper, S., Kikinis, R., 2012. 3D Slicer as an Image Computing Platform for the Quantitative Imaging Network. Magn Reson Imaging 30, 1323–1341. https://doi.org/10.1016/j.mri.2012.05.001

Solomon, O., Palnitkar, T., Patriat, R., Braun, H., Aman, J., Park, M.C., Vitek, J., Sapiro, G., Harel, N., 2021. Deep-learning based fully automatic segmentation of the globus pallidus interna and externa using ultra-high 7 Tesla MRI. Human Brain Mapping 42, 2862–2879. https://doi.org/10.1002/hbm.25409
